# Spontaneous Hemothorax and Hemoptysis in Granulomatosis With Polyangiitis: A Case Report

**DOI:** 10.1002/ccr3.70645

**Published:** 2025-09-01

**Authors:** Paolo Scanagatta, Sara Cagnetti, Casimiro Eugenio Giorgetta, Francesco Inzirillo, Eugenio Ravalli, Giuseppe Naldi, Manuela Agozzino, Gianluca Ancona

**Affiliations:** ^1^ Division of Thoracic Surgery, Ospedale “Eugenio Morelli” ASST Valtellina e Alto Lario Sondalo Italy; ^2^ Division of Pathology ASST Valtellina e Alto Lario Sondalo Italy

**Keywords:** ANCA‐associated vasculitis, granulomatosis with polyangiitis, immunosuppression, lobectomy, spontaneous hemothorax, video‐assisted thoracoscopic surgery (VATS)

## Abstract

Spontaneous hemothorax is a rare but potentially life‐threatening manifestation of granulomatosis with polyangiitis (GPA). This case highlights the importance of considering vasculitis in unexplained hemothorax and underscores the role of early surgical intervention in preventing deterioration in immunosuppressed patients. Multidisciplinary management is crucial for timely diagnosis, treatment, and optimizing outcomes.

## Graphical abstract

Histologic lung section illustrating chronic vasculitis characteristic of GPA.

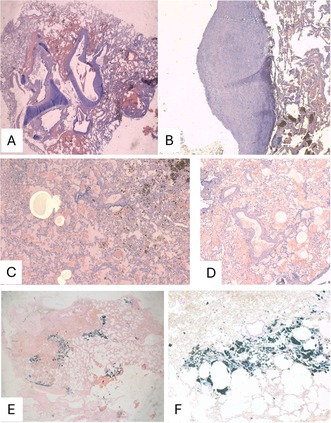

**Abbreviations**
AAV: ANCA‐associated vasculitides
ANCA: anti‐neutrophil cytoplasmic antibodies
AVPU: Alert, Verbal, Pain, Unresponsive
C‐ANCA: cytoplasmic anti‐neutrophil cytoplasmic antibodies
CRP: C‐reactive protein
CT: computed tomography
GBM: glomerular basement membrane
GPA: granulomatosis with polyangiitis
H&E: hematoxylin–eosin
ICU: Intensive Care Unit
MPO: myeloperoxidase
P‐ANCA: perinuclear anti‐neutrophil cytoplasmic antibodies
PR3: proteinase 3
SH: spontaneous hemothorax
VATS: video‐assisted thoracoscopic surgery

## 1 Introduction

Hemothorax is defined as an accumulation of blood in the space between the visceral and parietal pleura, which can occur secondary to penetrating or blunt trauma, coagulopathies, or iatrogenic complications (such as central venous catheter insertion, thoracentesis, or pleural biopsies) [1]. Spontaneous hemothorax (SH) is a subtype of hemothorax where blood accumulates in the pleural cavity in the absence of trauma or other obvious causes. The management of spontaneous hemothorax may involve both conservative and surgical approaches, with a particular focus on etiology‐specific treatments: Although pleural drainage is generally sufficient for treating hemothorax, in selected cases, video‐assisted thoracoscopic surgery (VATS) or thoracotomy may be required to achieve prompt, effective, and definitive treatment [1]. Granulomatosis with polyangiitis (GPA) and other anti‐neutrophil cytoplasmic antibodies (ANCA)‐associated vasculitides (AAV) can present with a variety of pulmonary manifestations, ranging from nodules to diffuse alveolar hemorrhage. A summary of vasculitides and their pulmonary involvement is provided in Table 1.

**TABLE 1:** Summary of vasculitic diseases and their pulmonary manifestations.

Type	Characteristics/Ab	Lung manifestation
Small vessel		
Churg–Strauss syndrome (EGPA)	p‐ANCA (MPO) in 40% patients	Rarely, pulmonary capillaritis can cause alveolar hemorrhage
Henoc–Schönlein Purpura	IgA‐mediated (IgAV)	IgAV without evidence of systemic vessel vasculitis
Idiopathic mixed cryoglobulinemia	CGs	Rarely, acute alveolar hemorrhage, organizing pneumonia, pulmonary vasculitis and pleural effusions in 5% of patients
Wegener's granulomatosis (GPA)	MPO‐ or PR3‐ANCA	Lung is involved in 70%–100% of patients
Microscopic polyangiitis (MPA)	ANCA directed against LAMP‐2, MPO‐ANCA	Diffuse alveolar hemorrhage caused by pulmonary capillaritis, which has been reported in 12%–55%
Medium vessel		
Kawasaki arteritis	Virus‐linked IgAV and IgAN	Lung involved rarely; coronary vasculitis
Polyarteritis nodosa (PAN), HBV‐PAN, Associated with ANCA vasculitis	Deficiency of ADA2	Pulmonary involvement is uncommon
Buerger's disease (TAO)	Unknown	Occlusion in distal extremities vessel
Big vessel		
Horton's temporal arteritis (GCA)	Associated with PMR	Pulmonary involvement, especially pleural involvement, is considered to be rare
Takayasu's arteritis	HLA complex association, AECA	Pulmonary involvement is uncommon
Polymyalgia rheumatica (PMR)	ESR and CRP; not CPK or LDH	Interstitial Lung Disease

Abbreviations: ADA2, adenosine deaminase‐2; AECA, Anti‐endothelial cell antibodies; CGs, Cryoglobulins; CPK, Creatine Phosphokinase; CRP, C‐reactive protein; EGPA, Eosinophilic granulomatosis with polyangiitis; ESR, Erythrocyte sedimentation rate; GCA, Giant cell arteritis; GPA, Granulomatosis with polyangiitis; HLA, Human leukocyte antigens; IgA, Immunoglobulin A; IgAN, IgA nephropathy; IgAV, IgA vasculitis; LAMP‐2, Lysosomal membrane protein‐2; LDH, Lactic dehydrogenase; MPO, Myeloperoxidase; p‐ANCA, Anti‐neutrophil cytoplasmic antibodies; PMR, Polymyalgia rheumatica; PR3, Proteinase 3; TAO, Thromboangiitis obliterans.

In this report, we present a case of spontaneous hemothorax following an infarction of the right lower lobe in a patient with granulomatosis with polyangiitis (GPA), along with a brief literature review. The case is presented in accordance with CARE guidelines [2].

## 2 Case History/Examination

A 67‐year‐old non‐smoking woman presented to the Emergency Department with asthenia and dyspnea. Her medical history included an episode of hemoptysis and acute renal failure, which had been managed with a kidney transplant in 2003 due to AAV with secondary glomerulonephritis and hemorrhagic alveolitis. Since then, the patient had been on chronic immunosuppressive therapy, including tacrolimus (2 mg/day), prednisone (5 mg/day), and mycophenolate mofetil (720 mg twice daily). She had tolerated long‐term immunosuppressive therapy well, without any significant adverse effects over the years.

Upon arrival, the level of consciousness was assessed as “Alert” using the Alert, Verbal, Pain, Unresponsive (AVPU) scale. Vital signs showed a respiratory rate of 30 breaths per min, a heart rate of 110 beats per min, and a blood pressure of 80/60 mmHg. Blood tests revealed severe anemia (hemoglobin 6 g/dL) despite a normal coagulation profile.

**FIGURE 1:**
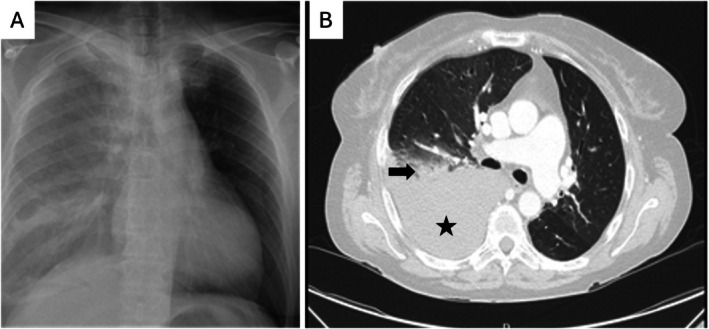
(A) Chest X‐ray showing a large right‐sided pleural effusion. (B) Chest CT scan demonstrating consolidation in the lower right lung (black arrow) and a concomitant hematoma (black star).

A chest X‐ray (Figure 1A) showed a large right‐sided pleural effusion, suggesting hemothorax. A subsequent CT scan (Figure 1B) revealed a right lower lobe infarction with an adjacent pleural hematoma, without evidence of pulmonary nodules or cavitations.

**FIGURE 2:**
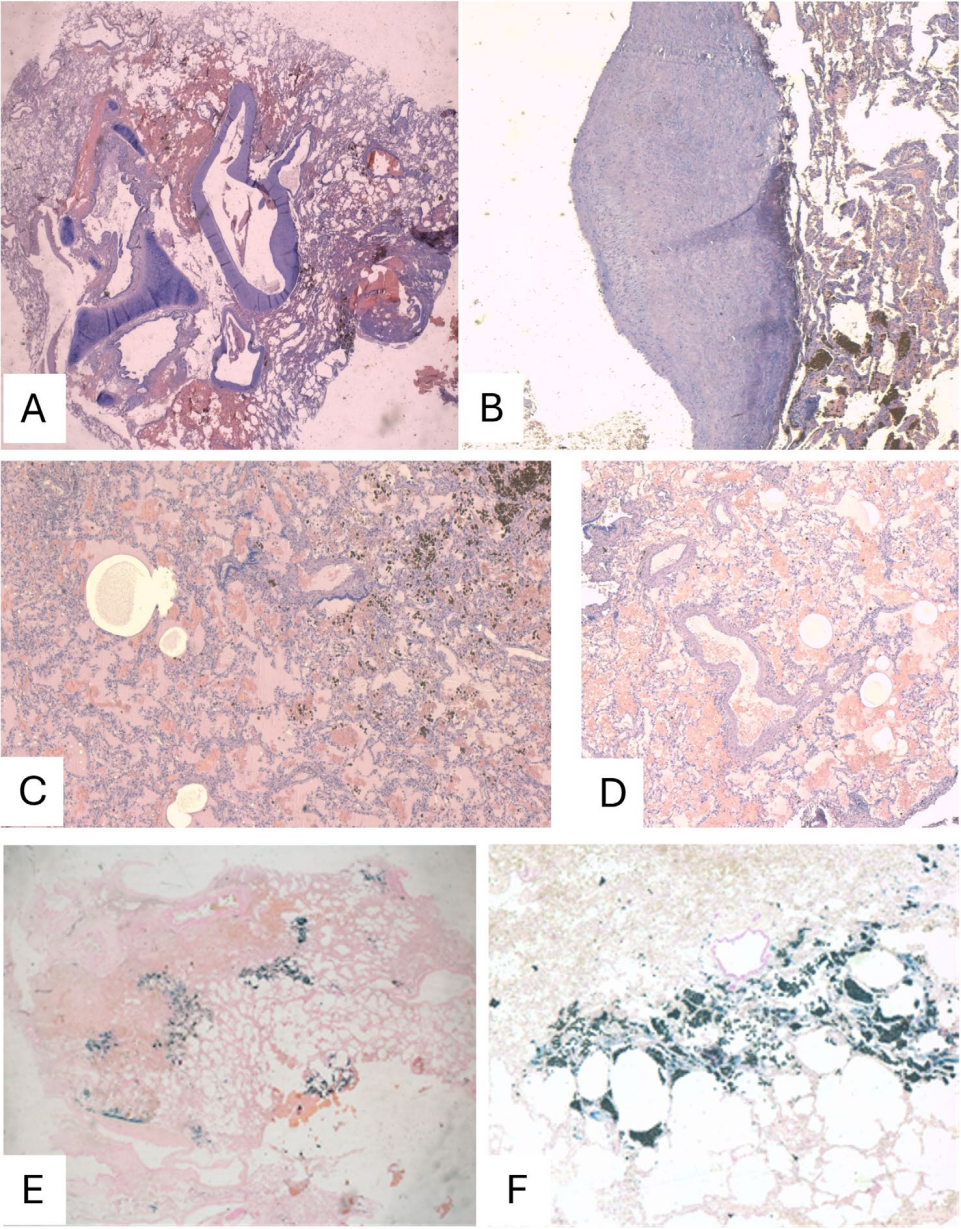
Histopathological analysis with hematoxylin–eosin (H&E) and Perls' Prussian Blue stains. (A) Hematoxylin–eosin (H&E) stain, original magnification ×2: Histological overview with a vessel showing intimal hyperplasia. (B) H&E stain, original magnification ×10: Magnification of the vessel from panel (A), showing eccentric intimal hyperplasia. (C) H&E stain, original magnification ×10: Parenchymal section with vessels affected by intimal hyperplasia and recent intra‐alveolar hemorrhage; black deposits indicate hemosiderin‐laden macrophages and hemosiderin pigment. (D) H&E stain, original magnification ×2: Additional parenchymal section with vessels involved in intimal hyperplasia and recent intra‐alveolar hemorrhage. (E) Perls' Prussian Blue stain, original magnification ×2: Detection of hemosiderin deposits (blue staining). (F) Perls' Prussian Blue stain, original magnification ×10: Higher magnification confirms the presence of hemosiderin‐laden macrophages and deposits.

Due to the initial hemorrhagic shock, an urgent VATS was performed. During the procedure, a large parenchymal hematoma was identified, necessitating a right lower lobectomy. Histopathological analysis ruled out malignancies or vascular malformations, instead revealing findings consistent with chronic vasculitis associated with GPA. The findings included intimal hyperplasia, myxoid degeneration of the vessels, and positivity for Perls' Prussian Blue stain (Perls+), with no evidence of active vasculitis or vascular infiltration (Figure 2).

**FIGURE 3:**
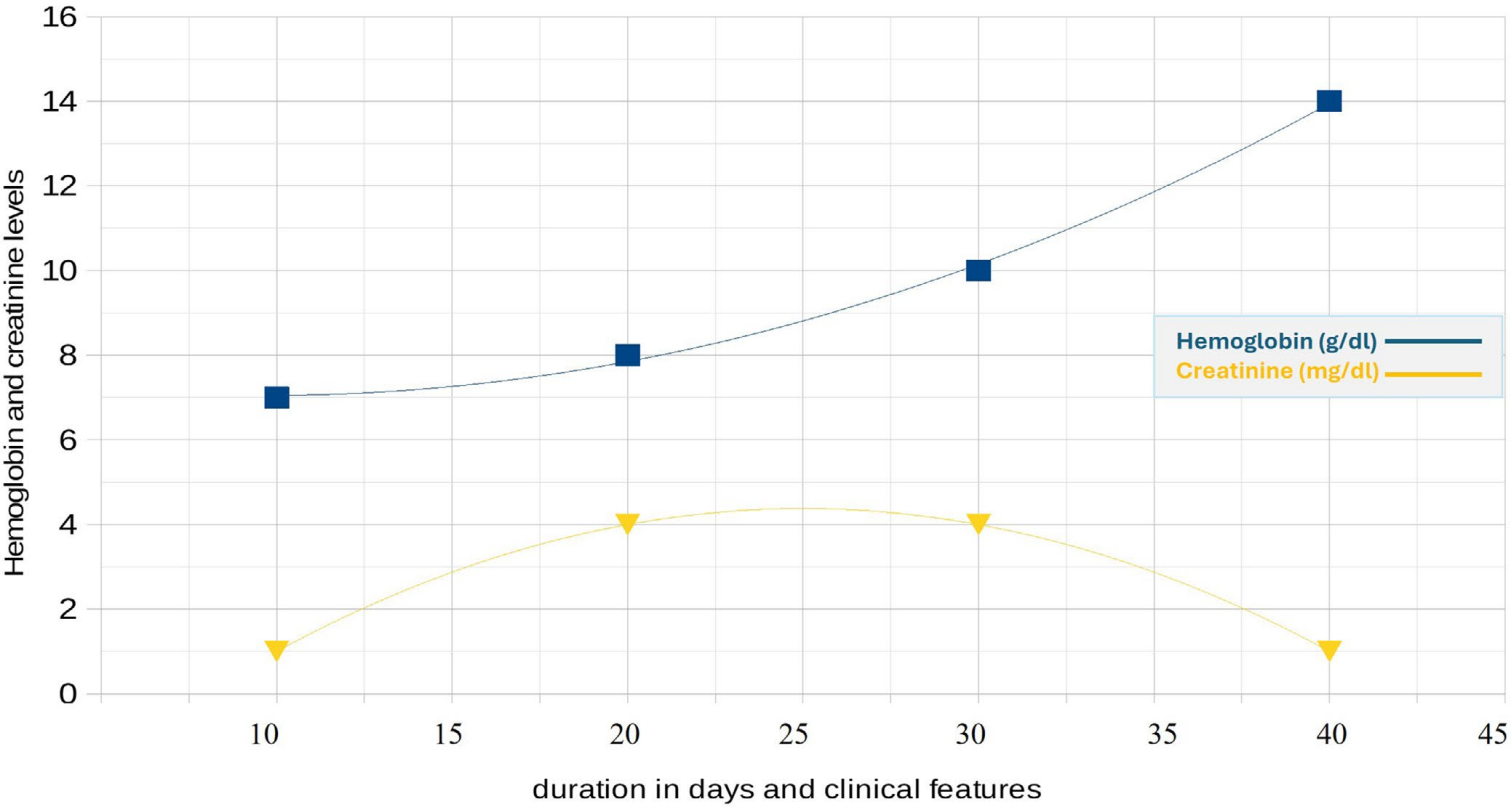
Case report timeline and laboratory trends: The blue line indicates hemoglobin levels (g/dL), and the yellow line indicates creatinine levels (mg/dL) over time. *Y*‐axis units are provided for both parameters. Case report timeline: Hemoptysis and alveolar hemorrhage (Days 1–5), pulmonary‐renal syndrome and leukocytoclastic vasculitis (Days 5–6), rapid deterioration and Intensive Care Unit (ICU) admission (Days 7–8), development of GPA (Days 9–39), stable clinical condition (Days 40–45).

Serological tests showed the presence of anti‐myeloperoxidase antibodies (anti‐MPO), associated with perinuclear anti‐neutrophil cytoplasmic antibodies (P‐ANCA). Tests for anti‐glomerular basement membrane antibodies (anti‐GBM) and cytoplasmic anti‐neutrophil cytoplasmic antibodies (C‐ANCA) specific for proteinase 3 (Anti‐PR3) were negative. During the hospital stay, creatinine levels remained stable, with no evidence of new renal impairment. The patient was subsequently transferred to the rheumatology department for continued management. A timeline summarizing the patient's clinical course, from initial presentation to diagnosis, surgical management, and follow‐up, is depicted in Figure 3.

## 3 Methods (Differential Diagnosis, Investigations, and Treatment)

### 3.1 Differential Diagnosis

The initial differential diagnosis included the following:

1. Malignancy‐related hemothorax—Excluded by histopathology, which showed no malignancy or vascular malformations.
2. Coagulation disorders—Normal coagulation parameters ruled out clotting abnormalities.
3. Connective tissue disorders—No serological evidence of systemic lupus erythematosus (SLE) or other connective tissue diseases.
4. ANCA‐associated vasculitis—Confirmed by persistent MPO‐ANCA positivity and histological findings.

### 3.2 Investigations

Upon admission, laboratory investigations revealed severe anemia (hemoglobin 6 g/dL) with a normal coagulation profile. Serological tests showed positive MPO‐ANCA at 20.3 UI/mL, with negative C‐ANCA and anti‐GBM antibodies. Chest X‐ray and CT scan findings are illustrated in Figure 1. Histopathology confirmed chronic vasculitis without active lesions.

### 3.3 Treatment

An urgent VATS procedure with right lower lobectomy was performed due to hemorrhagic shock and failure of conservative management. The patient received supportive care, transfusions, and continued her immunosuppressive regimen during the postoperative period.

## 4 Conclusions and Results (Outcome and Follow‐Up)

The patient had an uneventful postoperative recovery and remained hemodynamically stable. During the hospital stay, creatinine levels remained stable, with no signs of renal impairment. The patient was subsequently transferred to the rheumatology department for continued management. She was discharged with a personalized immunosuppressive regimen and regular follow‐ups in the rheumatology department. A timeline summarizing the patient's clinical course, from initial presentation to diagnosis, surgical management, and follow‐up, is depicted in Figure 3.

At the 6‐month follow‐up, the patient reported no recurrence of hemothorax or respiratory symptoms. Serological markers for disease activity remained stable, with MPO‐ANCA still positive at 20.3 UI/mL (normal range 0–5.97 UI/mL) but there were no new vasculitic flares. Imaging showed no evidence of new pulmonary lesions.

At the 12‐month follow‐up, she continued to be asymptomatic, maintaining stable respiratory function and immunosuppressive therapy without significant adverse effects. There were no signs of recurrence of hemothorax, alveolar hemorrhage, or extrapulmonary disease progression.

At the 22‐month follow‐up, P‐ANCA was still positive, but the patient remained in good general health, with stable kidney function and no new systemic manifestations of vasculitis. Moreover, following pulmonary surgery, the patient continued to tolerate immunosuppressive treatment without any new complications or side effects.

Throughout the follow‐up period, the patient reported being fully satisfied with the treatment outcome, providing a rating of 5 out of 5 on a 5‐point scale.

## 5 Discussion

GPA, formerly referred to as Wegener's granulomatosis, is a systemic necrotizing vasculitis primarily involving small blood vessels. First recognized in 1937 by Friedrich Wegener [3], GPA predominantly affects the upper and lower respiratory tract as well as the kidneys [4]. The classic triad of GPA includes granulomatous inflammation of the respiratory tract, pauci‐immune glomerulonephritis, and systemic vasculitis [5]. However, pulmonary manifestations can vary, including nodules, cavitations, infiltrates, and, more rarely, diffuse alveolar hemorrhage [6].

Spontaneous hemothorax is a rare entity, with only a few cases reported in the literature related to vasculitic diseases such as GPA [7]. While hemothorax is commonly associated with trauma, coagulation disorders, or invasive procedures, its spontaneous occurrence raises suspicion of an underlying vasculitic process or vascular fragility secondary to prolonged immunosuppressive therapy [8]. In our case, the patient, who had been on long‐term immunosuppression following a kidney transplant, presented with massive hemothorax without evident signs of diffuse alveolar hemorrhage or renal involvement.

The literature indicates that, in the presence of AAV, such as GPA, life‐threatening pulmonary hemorrhages can occur [9]. AAV are characterized by antibodies directed against MPO and PR3, associated with P‐ANCA and C‐ANCA phenotypes, respectively [10]. However, in our case, the absence of renal involvement and diffuse alveolar hemorrhage made the diagnosis of GPA less apparent, necessitating a combination of imaging, surgical intervention, and histopathology to confirm the clinical picture.

The decision to perform VATS and a right lower lobectomy was based on the patient's hemodynamic instability and the inability to control the bleeding with chest drainage alone. Surgical resection enabled effective hemorrhage control, preventing further complications. Histopathological analysis played a crucial role, revealing a pattern of chronic vasculitis without evidence of active vasculitis, consistent with prolonged immunosuppressive therapy [11].

The absence of acute vasculitic changes in the histopathological examination may be attributed to the patient's prolonged immunosuppressive therapy, which could have masked the inflammatory process. Furthermore, the infarction of the lower lobe due to massive hemothorax may have contributed to the histological alterations, making the vasculitic component less evident.

Few cases of spontaneous hemothorax in GPA patients have been reported in the literature, primarily in younger patients or during pregnancy [6]. Table 2 summarizes previously reported cases, emphasizing the rarity and variability of this presentation. However, the occurrence of pleural hemorrhage in an elderly patient with long‐term immunosuppression and isolated pulmonary involvement—without renal manifestations, contrary to what is typically reported in the literature [12]—represents an atypical clinical scenario that warrants attention.

**TABLE 2:** Case reports on spontaneous hemothorax in patients with Wegener's disease.

Case	Age, gender (years, M/F)	Side; symptoms	Comorbidity	Treatment	Operative findings	Follow up (months)
Serhane et al. [6]	51, F	Right; no symptoms	EGPA	FBO	MLS	1
Maranini et al. [7]	18, F	Left; dyspnea cough, chest pain	Pregnancy	Explorative Thoracotomy	Hemothorax 1000 mL	1
Scanagatta et al. (present case report)	67, F	Right; asthenia, dyspnea	Hemoptysis, acute renal failure, kidney transplant	VATS‐ Right lower lobectomy	Hemothorax 1000 mL, lung hematoma	22

Abbreviations: EGPA, Eosinophilic granulomatosis with polyangiitis; FBO, Operative fibro‐bronchoscopy; MLS, Middle lobe syndrome; VATS, Video‐assisted thoracoscopic surgery.

This case highlights the importance of early surgical intervention in spontaneous hemothorax associated with GPA. A multidisciplinary approach involving thoracic surgeons, rheumatologists, and pathologists was essential in achieving a favorable outcome. The rarity of this presentation underscores the need for increased awareness of vasculitis as a potential cause of spontaneous hemothorax in immunosuppressed patients. Early recognition and timely intervention are crucial in reducing mortality and enhancing the quality of life for affected patients.

## Author Contributions

**Paolo Scanagatta:** conceptualization, data curation, formal analysis, investigation, methodology, project administration, resources, software, supervision, validation, visualization, writing – original draft, writing – review and editing. **Sara Cagnetti:** writing – original draft, writing – review and editing. **Casimiro Eugenio Giorgetta:** writing – original draft, writing – review and editing. **Francesco Inzirillo:** writing – original draft, writing – review and editing. **Eugenio Ravalli:** writing – original draft, writing – review and editing. **Giuseppe Naldi:** writing – original draft, writing – review and editing. **Manuela Agozzino:** data curation, formal analysis, investigation, methodology, software, writing – original draft, writing – review and editing. **Gianluca Ancona:** conceptualization, data curation, investigation, methodology, software, visualization, writing – original draft, writing – review and editing.

## Consent

Written informed consent was obtained from the patient for the publication of this case report, including clinical data and images.

## Conflicts of Interest

The authors declare no conflicts of interest.

## Data Availability

The data that support the findings of this study are available on request from the corresponding author. The data are not publicly available due to privacy or ethical restrictions.
